# Supplementary material for: Indian Medicinal Plant-Derived Phytochemicals as Potential Antidotes for Snakebite: A Pharmacoinformatic Study of Atrolysin Inhibitors
Source: Int J Mol Sci. 2024 Nov 26;25(23):12675. doi: 10.3390/ijms252312675 (PMC11641534; doi:10.3390/ijms252312675)
Supplement: Supplementary file 1 [file ijms-25-12675-s001.zip › Supplementary.pdf]

International Journal of
Molecular Sciences

MDPI

Supplementary Materials

Indian Medicinal Plant-Derived Phytochemicals as Potential Antidotes for Snakebite: A Pharmacoinformatic Study of Atrolysin Inhibitors

Deva Asirvatham Ravi [1], Du Hyeon Hwang [1,2], Ramachandran Loganathan Mohan Prakash [1], Changkeun Kang [1,2] and Euikyung Kim [1,2,*]

[1] College of Veterinary Medicine, Gyeongsang National University, Jinju 52828, Republic of Korea; devabiochem@gnu.ac.kr (D.A.R.); pooh9922@gnu.ac.kr (D.H.H.); mohanprakash111@gmail.com (R.L.M.P.); ckkang@gnu.ac.kr (C.K.)

[2] Institute of Animal Medicine, Gyeongsang National University, Jinju 52828, Republic of Korea

* Correspondence: ekim@gnu.ac.kr; Tel.: +82-55-772-2355; Fax: +82-55-772-2349

Citation: Ravi, D.A.; Hwang, D.H.; Mohan Prakash, R.L.; Kang, C.; Kim, E. Indian Medicinal Plant-Derived Phytochemicals as Potential Antidotes for Snakebite: A Pharmacoinformatic Study of Atrolysin Inhibitors. *Int. J. Mol. Sci.* **2024**, *25*, 12675. https://doi.org/10.3390/ijms252312675

Academic Editors: José Marco-Contelles and Cleydson Breno Rodrigues dos Santos

Received: 11 October 2024
Revised: 19 November 2024
Accepted: 23 November 2024
Published: 26 November 2024

Copyright: © 2024 by the authors. Licensee MDPI, Basel, Switzerland. This article is an open access article distributed under the terms and conditions of the Creative Commons Attribution (CC BY) license (https://creativecommons.org/licenses/by/4.0/).

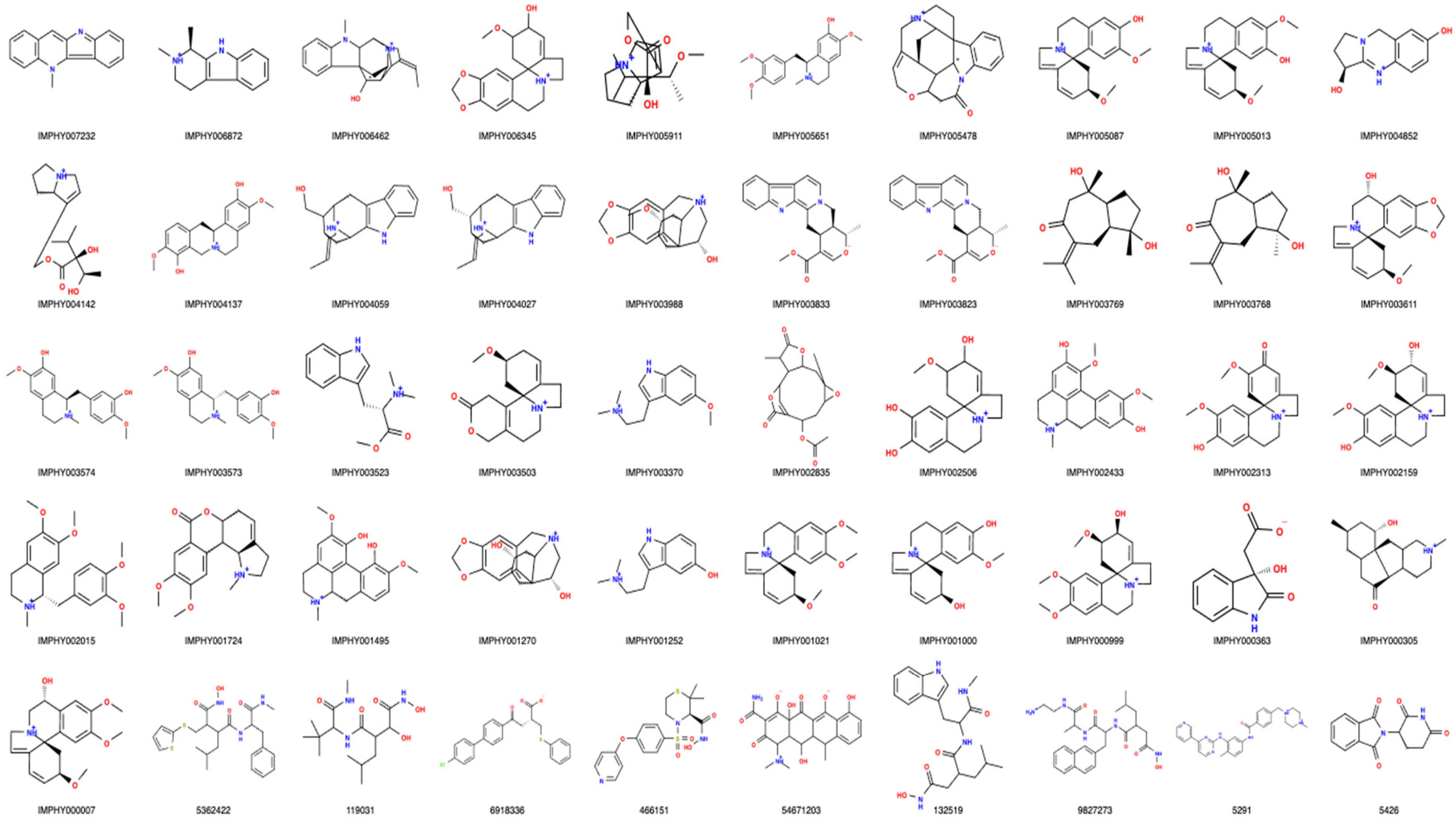
IMPHY007232
IMPHY006872
IMPHY006462
IMPHY006345
IMPHY005911
IMPHY005651
IMPHY005478
IMPHY005087
IMPHY005013
IMPHY004852
IMPHY004142
IMPHY004137
IMPHY004059
IMPHY004027
IMPHY003988
IMPHY003833
IMPHY003823
IMPHY003769
IMPHY003768
IMPHY003611
IMPHY003574
IMPHY003573
IMPHY003523
IMPHY003503
IMPHY003370
IMPHY002835
IMPHY002506
IMPHY002433
IMPHY002313
IMPHY002159
IMPHY002015
IMPHY001724
IMPHY001495
IMPHY001270
IMPHY001252
IMPHY001021
IMPHY001000
IMPHY000999
IMPHY000363
IMPHY000305
IMPHY000007
5362422
119031
6918336
466151
54671203
132519
9827273
5291
5426

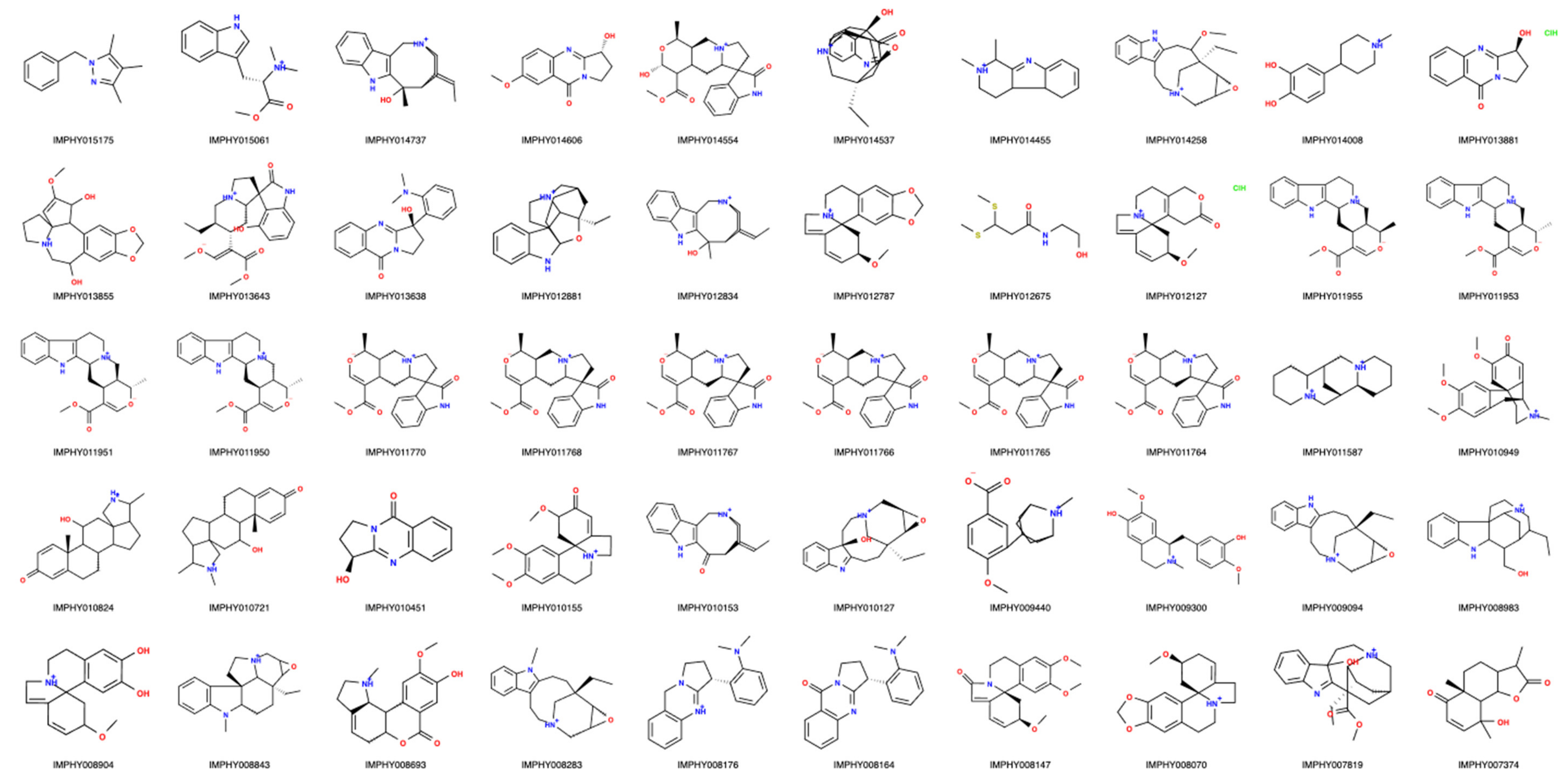

Supplementary .Fig. S1 (cont.)

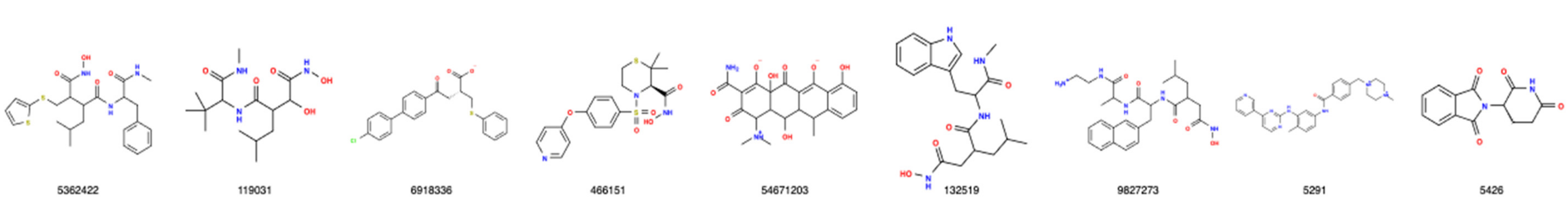

Supplementary Figure S1. The chemical structure of the selected compound.

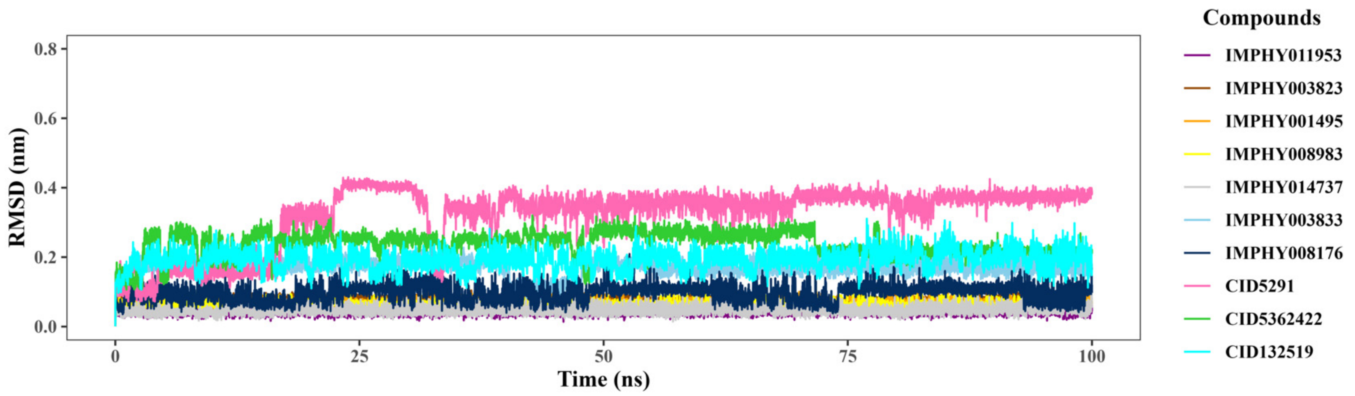

Supplementary Figure S2. Ligand RMSD of the compound was recorded for 100ns.

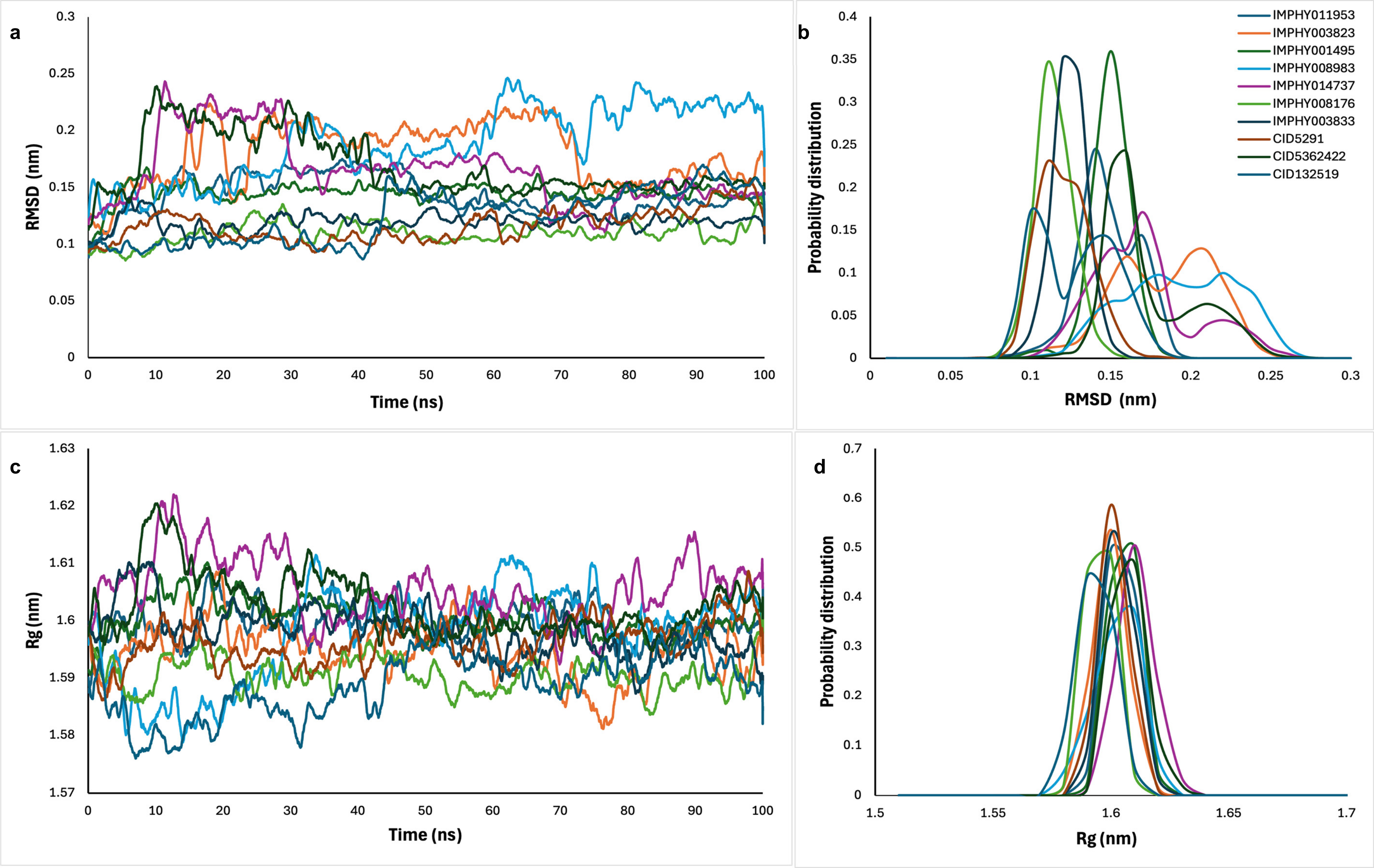
a
RMSD (nm)
Time (ns)
b
Probability distribution
RMSD (nm)
IMPHY011953
IMPHY003823
IMPHY001495
IMPHY008983
IMPHY014737
IMPHY008176
IMPHY003833
CID5291
CID5362422
CID132519
c
Rg (nm)
Time (ns)
d
Probability distribution
Rg (nm)

Supplementary Figure S3. (a) Root Mean Square Deviation (RMSD) moving average for the complexes over 100 ns, (b) RMSD distribution plot for the complexes over 100 ns (c) Radius of Gyration moving average for the complexes over 100 ns , and (d) Radius of Gyration distribution plot for the complexes over 100 ns.
